# Mutant molecular chaperone activates cytokine receptor as a homomultimer

**DOI:** 10.18632/oncotarget.26221

**Published:** 2018-10-16

**Authors:** Marito Araki, Norio Komatsu

**Affiliations:** Norio Komatsu: Department of Hematology, Juntendo University Graduate School of Medicine, Tokyo, Japan

**Keywords:** neoplasms, CALR, MPL, JAK2

Sustenance of proliferation signaling is one of the hallmarks of cancer. In myeloproliferative neoplasms (MPNs), one or more lineages of myeloid cells proliferate excessively, due to oncogenic transformation of hematopoietic stem/progenitor cells exhibiting cytokine hypersensitivity. Consistent with the cell biological features observed in MPN, gain-of-function mutations on *Myeloproliferative Leukemia Protein (MPL) and Janus Kinase 2 (JAK2)*, encoding a thrombopoietin receptor and its downstream tyrosine kinase, respectively, were previously identified as driver mutations for MPN [1]. In 2013, recurrent frameshift mutations in *calreticulin (CALR)* encoding an ER-residing molecular chaperone were discovered to be present in a mutually exclusive manner with the *JAK2* and *MPL* mutations in patients with MPN [2, 3], suggesting an oncogenic role of mutant CALR in the MPL-JAK2 axis. Indeed, our group and others have demonstrated that mutant CALR constitutively activates MPL through its preferential binding to MPL, thus promoting hematopoietic cell transformation [4-7].

**Figure 1:**
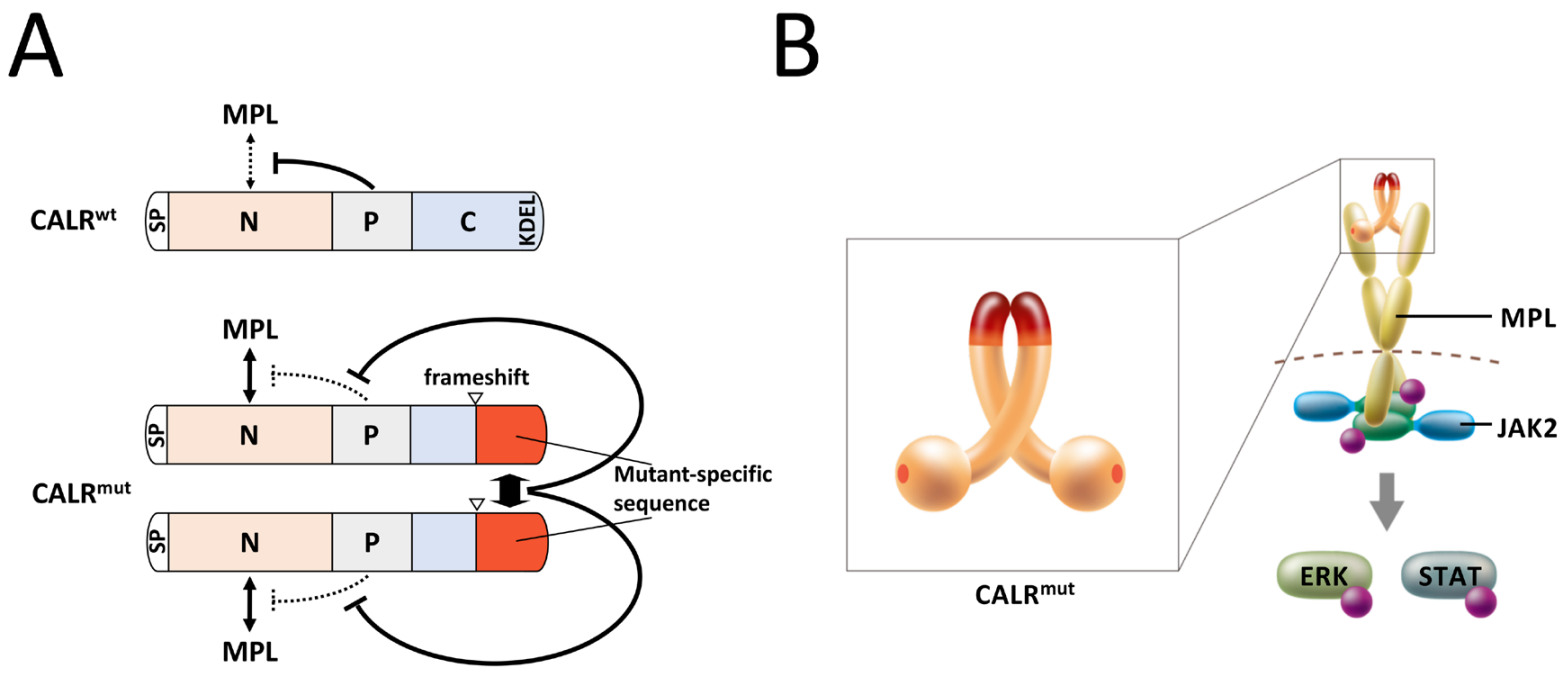
A model for the mutant CALR-dependent MPL activation **A.** Domain structures of wild-type (CARL^wt^) and mutant (CALR^mut^) CALR adopted and modified from ref 8. CALR proteins consist of the following domains: a signal peptide (SP), amino-terminal N-domain (N), proline rich P-domain (P), carboxy-terminal C-domain (C) that includes an endoplasmic reticulum retention signal, KDEL, in the wild type. The P-domain blocks binding of the N-domain to MPL in wild-type CALR [4]. Arrowheads indicate the boundary between the amino acid sequences unaffected and affected by the frameshift mutation. Owing to the frameshift mutation, CALR^mut^ loses a portion of C-domain and gains a mutant-specific sequence common to all types of CALR^mut^. The domains with mutant-specific sequences interact with each other to form a homomultimeric complex, which induces a presumptive structural change to block the P-domain and thus enables N-domain binding to MPL [4, 10]. **B.** A model for the constitutive activation of MPL by a dimerized CALR^mut^ adopted and modified from ref 10. Homomultimeric mutant CALR binding presumably induces a structural change in predimerized MPL and thus activates JAK2. Although the model shows a mutant CALR homodimer, more than two molecules of mutant CALR could form homomultimeric complexes [10].

*CALR* mutations found in MPN are unique: all of them are +1 frameshift mutations mapped to a very narrow region in the last exon of the gene, and change the carboxyl(C)-terminal sequence enriched with negatively charged amino acids in the wild-type protein to a cluster of positively charged amino acids (Figure 1A). We have previously demonstrated that the N-domain, localized in the amino-terminal region of CALR, interacted with MPL in a manner dependent on the mutant-specific domain in the C-terminal region of the mutant CALR [4], and proposed a model in which the mutant-specific domain induced a presumptive structural change that allows N-domain binding to MPL [8]. However, because three-dimensional structure of mutant CALR has not yet been solved, the nature of this structural change remains unknown.

One molecule of a cytokine harboring two receptor-binding sites is thought to simultaneously bind to two molecules of type-1 cytokine receptor that form a homodimer for its activation [9]. Therefore, we hypothesized that mutant CALR forms a homodimer, which confers mutant CALR two receptor-binding sites and thus triggers mutant CALR to interact with and activate homo-dimerized MPL. Through a series of biochemical characterization experiments, we have recently shown that mutant, but not wild-type CALR, de facto forms a homomultimeric complex [10]. The intermolecular interaction between mutant CALR was mediated by the mutant-specific-sequence (Figure 1A). Furthermore, we demonstrated that the intermolecular interaction was required for the binding and subsequent activation of MPL [10]. This implied that the previously proposed structural change dependent on mutant specific domain was induced by the intermolecular interaction between mutant CALR molecules, which causes the mutant CALR to interact with dimerized MPL for its activation. Collectively, we proposed a new model for the constitutive activation of cytokine receptor signaling by a mutant molecular chaperone, which serves as a fake ligand by forming a homomultimeric complex that simultaneously interacts with multiple receptor molecules, leading to the activation of downstream signaling molecules (Figure 1B).

Since we have demonstrated that inhibiting homomultimerization suppressed the ability of mutant CALR to interact with MPL and the subsequent oncogenic activation of MPL, inhibition of multimerization of mutant CALR is a potential therapeutic strategy to treat patients with CALR-positive MPN [10]. Nevertheless, further in-depth structural and cell biological studies regarding the oncogenic activation of cytokine receptors by mutant molecular chaperons are required to understand and target this unusual mechanism of cellular transformation.
